# Ultrasonic-Assisted Extraction and Natural Deep Eutectic Solvents Combination: A Green Strategy to Improve the Recovery of Phenolic Compounds from *Lavandula pedunculata* subsp. *lusitanica* (Chaytor) Franco

**DOI:** 10.3390/antiox10040582

**Published:** 2021-04-09

**Authors:** Inês Mansinhos, Sandra Gonçalves, Raquel Rodríguez-Solana, José Luis Ordóñez-Díaz, José Manuel Moreno-Rojas, Anabela Romano

**Affiliations:** 1 MED—Mediterranean Institute for Agriculture, Environment and Development, Faculdade de Ciências e Tecnologia, Campus de Gambelas, Universidade do Algarve, 8005-139 Faro, Portugal; ifmansinhos@ualg.pt (I.M.); raquel.rodriguez.solana@juntadeandalucia.es (R.R.-S.); 2 Department of Food Science and Health, Andalusian Institute of Agricultural and Fisheries Research and Training (IFAPA), Avenida Menendez-Pidal, SN, 14004 Córdoba, Spain; josel.ordonez@juntadeandalucia.es (J.L.O.-D.); josem.moreno.rojas@juntadeandalucia.es (J.M.M.-R.)

**Keywords:** natural deep eutectic solvents (NADES), ultrasound-assisted extraction (UAE), *Lavandula pedunculata*, phenolic compounds, phenolic acids, hydroxycinnamic acids, antioxidant activity, enzyme inhibition, HPLC, mass spectrometry

## Abstract

The present study aimed at evaluating the effectiveness of different natural deep eutectic solvents (NADES) on the extraction of phenolic compounds from *Lavandula pedunculata* subsp. *lusitanica* (Chaytor) Franco, on the antioxidant activity, and acetylcholinesterase (AChE), butyrylcholinesterase (BChE) and tyrosinase (Tyr) inhibitory capacities. Ten different NADES were used in this research and compared with conventional solvents. Ultrasound-assisted extraction (UAE) for 60 min proved to be the best extraction condition, and proline:lactic acid (1:1) and choline chloride:urea (1:2) extracts showed the highest total phenolic contents (56.00 ± 0.77 mg_GAE_/g_dw_) and antioxidant activity [64.35 ± 1.74 mg_TE_/g_dw_ and 72.13 ± 0.97 mg_TE_/g_dw_ in 2.2-diphenyl-1-picrylhydrazyl (DPPH) and 2.2′-azino-bis(3-ethylbenzothiazoline-6-sulfonic acid) (ABTS) methods, respectively]. These extracts also exhibited enzymes inhibitory capacity particularly against Tyr and AChE. Even so, organic acid-based NADES showed to be the best extractants producing extracts with considerable ability to inhibit enzymes. Twenty-four phenolic compounds were identified by HPLC-HRMS, being rosmarinic acid, ferulic acid and salvianolic acid B the major compounds. The results confirmed that the combination of UAE and NADES provide an excellent alternative to organic solvents for sustainable and green extraction, and have huge potential for use in industrial applications involving the extraction of bioactive compounds from plants.

## 1. Introduction

*Lavandula* is considered one of the most important genera in the vast vegetation cover of Mediterranean region. This genus belongs to the Lamiaceae family and comprises 39 species, many hybrids and nearly 400 cultivars [1,2]. Many of these plants have been cultivated in different regions, like Europe, North and South America, India, South West Asia, and the Arabian Peninsula [3]. *Lavandula* species have an interesting economic value as ornamentals and in several industry branches, like pharmaceutical, food, aromatherapy, perfumery and cosmetics, due to its essential oils [2,3]. Traditionally, in Portuguese folk medicine, some diseases (i.e., bronchitis, cough, anxiety, insomnia and anorexia) are treated with the consume of infusions prepared from flowered aerial parts of these plants [3,4].

*L. pedunculata*, can reach up to 70 cm tall, resists to annual variation in temperature and grows in altitudes up to 1700 m, being considered the most resistant *Lavandula* species [3,5]. Several reports showed that this species produces bioactive compounds with biological properties, namely antioxidant, antitumor, anti-inflammatory and antimicrobial activities [5,6,7]. According to the *Nova Flora de Portugal* [8], three *L. pedunculata* subspecies are distinguished in Portugal: subsp. *pedunculata* (northwest), subsp. *sampaiana* (north and central), and subsp. *lusitanica* (central and south). Costa et al. [4] previously identified phenolic compounds such as rosmarinic, 3-O-caffeoylquinic, 4-O-caffeoylquinic, 5-O-caffeoylquinic acids, luteolin, and apigenin in extracts from the subspecies *lusitanica* Franco, as well as demonstrated their antioxidant and anti-acetylcholinesterase potential. Phenolic compounds are recognized for their therapeutic capabilities for human diseases reducing, for example, the risk of cancer, diabetes, and cardiovascular and neurodegenerative pathologies. Among their numerous biological properties, the antioxidant characteristics of phenolics are responsible for a great part of the protective effects, namely decreasing the reactive oxygen species levels associated with these conditions [9]. Phenolic compounds have an aromatic ring bearing at least one hydroxyl substituents and depending on the number of phenol rings and their elements attached, these molecules may be classified in phenolic acids, flavonoids, coumarins, stilbenes, tannins, and lignans [5,10].

The solvents currently used in the extraction processes in pharmaceutical, nutraceutical, perfume, cosmetic and food ingredients industries have petroleum origin. These conventional organic solvents, obtained from non-renewable resources, have a great extraction power and dissolution ability and, therefore, are still extensively used to extract natural compounds as antioxidants. However, their use is known to be harmful to both human health and environment [11]. To overcome these constraints, in 2015 emerged a plan outlined by United Nations—“Transforming Our World: The 2030 Agenda for Sustainable Development”—that addresses a wide range of issues, many of which recognize the need of green and sustainable tools to protect the planet from environmental degradation [12]. Green solvents appear as an alternative to organic solvents given their advantageous attributes, namely decreasing the pollution impact and energy usage in extractions, and the obtained extracts can be safely used in food, pharmaceutical and cosmetic industries [11,13].

In recent years, a new class of green solvents—deep eutectic solvents (DES)—has attracted a lot of attention. These solvents are characterized as a mixture of two or more components—a hydrogen bond acceptor (HBA) and a hydrogen bond donor (HBD)— that have a much lower melting point than that of any of their individual components. The combination of components upon mixing in particular molar ratios leads to a mixture with extremely lower melting temperature, close to the ambient temperature [14,15]. When DES are prepared with components with natural origin such as choline chloride, organic acids, carbohydrates, and amino acids, they are called natural deep eutectic solvents (NADES). These are considered more environmentally friendly, biodegradable, and non-toxic and therefore, they can be used without danger for human health in many industries, namely food, pharmaceutical and cosmetic [11,13]. The interaction of hydrogen bonding between the HBAs and the HBDs is the main force required to produce NADES. Besides having significant influence on physicochemical properties such as viscosity, density, conductivity, polarity and solubilization ability, NADES composition can affect the extraction efficiency of target compounds [14,16]. In combination with several innovative extraction techniques, these eco-friendly solvents have been recently used to extract, separate and pre-concentrate bioactive compounds from natural sources, namely polyphenols (e.g., phenolic acids and flavonoids), alkaloids, cannabinoids, ginkgolides, etc. [17,18]. Besides NADES composition and molecular structure, other factors like molecular ratio, water content, temperature, extraction time, solvent/sample ratio, and pH play an important role in the efficiency and yield of the extraction process [15]. 

Conventional extraction techniques, like maceration, are usually associated with elevated organic solvent consumption and long extraction times. Ultrasound-assisted extraction (UAE) emerges as a good alternative to establish an environmentally friendly extraction method. It involves physical and chemical phenomena that are completely different from those applied in conventional extraction techniques. The propagation of ultrasound pressure waves and resulting cavitation forces induce the explosively collapse of bubbles and generate localized pressure causing plant tissue rupture and improving the release of intracellular substances into the solvent [18,19]. Some of the advantages of this methodology are mainly related to the reduced solvent consumption and energy requirements [20].

In the present study, different NADES were investigated for its capacity to extract bioactive compounds from *Lavandula pedunculata* subsp. *lusitanica* (Chaytor) Franco, using UAE and maceration as extraction procedures. To the best of our knowledge, this is the first study investigating the antioxidant properties of *L. pedunculata* using green solvents and also the first report evaluating acetylcholinesterase (AChE) and butyrylcholinesterase (BChE) inhibition capacity of NADES-based plant extracts. It is also the first evaluation of tyrosinase (Tyr) inhibition capacity of *L. pedunculata*. The phenolics in the extracts were identified and quantified by high performance liquid chromatography-high resolution mass spectrometry (HPLC-HRMS), the antioxidant activity evaluated using different methods, and the capacity to inhibit AChE, BChE and Tyr enzymes were also investigated.

## 2. Materials and Methods

### 2.1. Chemicals and Reagents

Trichloroacetic acid (TCA), 2,2′-azino-bis (3-ethylbenzothiazoline-6-sulfonic acid) diammonium salt tablets (ABTS), sodium phosphate dibasic anhydrous (Na_2_HPO_4_), acetylthiocholine iodide (ATCI), S-butyrylthiocholine iodide (BTCI), 5,5′-dithiobis(2-nitrobenzoic acid) (DTNB), 2,2-diphenyl-1-picrylhydrazyl (DPPH), acetylcholinesterase from Electrophorus electricus (AChE) (Electric-eel, EC 3.1.1.7, Type VIS), horse serum butyrylcholinesterase (BChE) (EC 3.1.1.8), mushroom tyrosinase (Tyr) (EC 1.14.18.1), 3,4-dihydroxy-L-phenylalanine (L-DOPA), potassium persulfate (K_2_S_2_O_8_), galanthamine, kojic acid, glucose, xylitol (99%), glycerol (99%), HPLC-MS-grade acetonitrile, HPLC-MS-grade water, formic acid, luteolin and chlorogenic acid were purchased from Sigma–Aldrich (Steinheim, Germany). Ethanol absolute for analysis, methanol for analysis and urea were obtained from Fisher Scientific (Leicestershire, UK). Dipotassium hydrogen phosphate (K_2_HPO_4_), citric acid (>99.5%), and naringenin were acquired from Fluka (Buchs, Switzerland). Potassium dihydrogen phosphate (KH_2_PO_4_) and ascorbic acid were provided by Merck (Darmstadt, Germany). Sodium dihydrogen phosphate monohydrate (NaH_2_PO_4_·H_2_O), fluorescein and lactic acid for analysis were obtained from Panreac (Barcelona, Spain). Folin-Ciocalteu’s phenol reagent (F-C reagent), gallic acid, sodium carbonate anhydrous (Na_2_CO_3_) and ferric chloride (FeCl_3_) were purchased from VWR (Leuven, Belgium). Potassium ferricyanide (K_3_[Fe(CN)_6_]), (±)-6-hydroxy-2,5,7,8-tetramethylchromane-2-carboxylic acid (Trolox), 2,2′-azobis(2-methylpropionamidine)dihydrochloride) (AAPH), choline chloride (99%), malic acid (>99.5%) and L-proline (>99%) were acquired from Acros Organics (Geel, Germany). Ferulic acid, caffeic acid, apigenin and ρ-coumaric acid were supplied by AASC Ltd. (Southhampton, UK), and rosmarinic acid was provided by Extrasynthese (Genay, France).

### 2.2. Plant Material

Aerial parts (stems, leaves and flowers) of *L. pedunculata* subsp. *lusitanica* (Chaytor) Franco were collected in September 2018 at Campus de Gambelas (Faro, Algarve, south Portugal). A voucher specimen was deposited in the herbarium of the University of Algarve (ALGU 8080). The plant material was dried in an oven (40 °C) until constant weight, ground to powder (<2 mm particle size) and stored at −20 °C until used.

### 2.3. NADES Preparation

The preparation of NADES was based on the heating and stirring method reported by Bentley et al. [21]. The mixtures, with a known amount of distilled water to facilitate dissolution, were heated at 50–80 °C in a constant temperature heating magnetic stirrer. The synthesis time was adjusted to generate a homogenous transparent liquid. 

The high viscosity is the biggest problem of NADES [22], which leads to slow mass transfer in extractions and time-consuming solvent transfer operations. This constraint can be partly overcome by the addition of water in a fair proportion because an excessive water content is also not recommended [16,23]. It was previously established that 30% of water is the ideal percentage to enhance the extraction yield of bioactive compounds [22,23,24] and therefore in this work NADES were prepared with a final percentage of water of 30% (*w*/*w*). 

The different types of mixtures prepared and used in the extraction experiments, their abbreviated designations, the molar ratios of their components, and visual appearance are shown in Table 1.

### 2.4. Extraction Procedure

The plant material was extracted in 100 mL Erlenmeyer flasks at 50 °C using two distinct techniques—maceration (M) and ultrasound-assisted extraction (UAE)—and different NADES combinations (Table 1) with a plant/solvent proportion of 0.25:10 *(w/v)*. With the aim to compare extraction efficiency of NADES and conventional solvents, water, ethanol 80% (EtOH 80) and methanol (MeOH) were also tested as extractant solvents. For maceration, the extraction was performed in a SW22 Shaking Water Bath (Julabo, Seelbach, Germany) at 200 rpm for 60 min. Regarding UAE, an Elmasonic S 100 H (220–240 V, 550 W) ultrasound bath (Elma Hans Schmidbauer GmbH & Co. KG, Singen, Germany) with 9 L of water was used at a frequency of 37 kHz (in sweep-function) at different extraction periods (15, 30 and 60 min). Since flask positioning in the ultrasound bath has been shown to affect the extraction efficiency [19], during extraction procedure all Erlenmeyer flasks were kept in the same position and the water was kept above the level of the solvent in the flasks. All extracts were filtered through Whatman nº. 1 filter paper (Whatman Int. Ltd., Maidstone, England) and the filtrates were stored at −20 °C until use.

### 2.5. Determination of Phenolic Compounds from the Extracts 

#### 2.5.1. Total Phenolics Contents (TPC) by Folin-Ciocalteu (F-C) Method

The total phenolic contents were determined by a spectrophotometric method which used Folin-Ciocalteu (F-C) reagent as described by Ainsworth and Gillespie [25]. F-C reagent 10% (*v*/*v*) (200 μL) was mixed with each extract diluted in phosphate buffer (75 mM, pH 7.0) (100 μL) and Na_2_CO_3_ (700 mM) (800 μL). After an incubation period of 2 h, at room temperature in the dark, the absorbance of the reaction mixture was measured at 765 nm. Gallic acid was used as standard and the results were expressed as milligrams of gallic acid equivalents per gram of dry weight (mg_GAE_/g_dw_), determined using a gallic acid standard curve (0.004–0.5 mM). 

#### 2.5.2. Phenolic Profile Analysis by HPLC-HRMS

The diluted plant extracts (1:4) were analyzed using a Dionex Ultimate 3000 HPLC system comprising of a HPLC pump and an autosampler operating at 4 °C (Thermo Scientific, San Jose, CA, USA). The injection volume was 5 μL, and the reverse phase separations were carried out using a 150 × 4.6 mm i.d. 5 μm 100 A C18 Kinetex column (Phenomenex, UK) maintained at 40 °C and eluted at a flow rate of 1.0 mL/min. The chromatographic conditions were carried out following those used by Gonçalves et al. [26] with slight modifications. The solvents used as the mobile phase were water (A) and acetonitrile (B), both with 0.1% formic acid. The gradient flow was as follows: 0 min—90% A; 10 min—74% A; 22 min—35% A; 30 min—5% A; 40 min—5% A; 40.1 min—90% A and 45 min—90% A. The column eluate was split, and 0.2 mL/min directed to an Exactive Orbitrap mass spectrometer (Thermo Fisher Scientific, CA, USA) fitted with a heated electrospray ionization probe (HESI) operating in negative ionization mode, scanning the ions in the *m/z* range from 100 to 1000. Full scans were recorded with a resolution of 50,000 and with a full automatic gain control (AGC) target of 1,000,000 charges, using 2 microscans. The analyses were also based on in-source collision-induced dissociation scans at 25 eV. The capillary temperature was 320 °C, the heater temperature was 150 °C, the sheath gas and the auxiliary gas flow rate were 25 and 5 units, respectively, and the spray voltage was 4.00 kV. Data acquisition and processing were carried out using Xcalibur software (Thermo Fisher Scientific, San José, CA, USA). The Exactive Orbitrap was externally calibrated weekly using ready-to-use calibration mixtures (Pierce ESI Negative Ion Calibration Solution and Pierce LQT ESI Positive Ion Calibration Solution, both available from Thermo Fisher Scientific, San José, CA, USA). A quality control (QC) sample was applied to assess and ensure that the analytical process was performed appropriately. The QC sample, composed of identical aliquots of a representative pool of the samples (plant extracts), was injected regularly throughout the run. This QC sample represented both the sample matrix and metabolite composition of the samples and was used to monitor drifts and to determine the variance of a metabolite feature (below 20%). 

Targeted identifications of phenolic compounds were achieved by comparing the exact mass and the retention time (RT) with available standards. In the absence of standards, compounds were tentatively identified by comparing the theoretical exact mass of the molecular ion with the measured accurate mass of the molecular ion and searched against Metlin, Phenol Explorer, PubChem and ChemSpider metabolite databases. In addition, these compounds were previously identified in plants of the same genus [1,27,28]. Metabolites having molecular masses within the pre-specified tolerance (mass difference less than 5 ppm) of the query masses are retrieved from these databases. Additionally, the identification of compounds was carried out following the MSI MS levels previously established by Sumner et al. [29], in which the metabolites identified using *m/z*, RT and/or MS/MS of reference standards were classified in level 1 and putatively annotated compounds using *m/z*, RT and/or MS/MS from spectral library and no reference standards were labelled in Level 2. The characteristics such as exact mass, delta ppm between experimental, retention time and MSI MI level are summarized in Supplementary Table S1. Quantification of compounds were carried out by selecting the theoretical exact mass of the molecular ion by reference to standard curves. In absence of reference compounds, they were quantified by reference to the calibration curve of a closely related parent compound (based on their structures). The linearity was determined for all the available standards. Limits of detection (LOD) and limits of quantification (LOQ) were estimated from the standard deviation of ten determinations of a blank. LOD and LOQ ranged from 0.00 to 0.49 mg/L and 0.01 to 1.64 mg/L, respectively. The different parameters used in quantification of phenolic compounds are summarized in Supplementary Table S2. All the analyses were performed in triplicate. 

### 2.6. Antioxidant Capacity

#### 2.6.1. DPPH Free Radical Scavenging Assay

Antioxidant activity was measured using the DPPH radical assay, as described by Soler-Rivas et al. [30] with slight modifications. DPPH methodology consists of the scavenger of the free radical DPPH^•^ by the action of an antioxidant. Thirty microliters of plant extract were added to 300 μL of 90 μM DPPH methanolic solution. The mixture was diluted with 570 µL of methanol 80% and after an incubation period of 30 min, the absorbance was read at 515 nm. Trolox (0.025–0.3 mM) was used as standard and the results were expressed as milligrams of Trolox equivalents per gram of dry weight (mg_TE_/g_dw_).

#### 2.6.2. ABTS Free Radical Scavenging Assay

Free radical scavenging activity of plant samples was also determined by ABTS radical cation decolorization assay described by Re et al. [31]. To produce ABTS radical cation, a stock solution of 7 mM ABTS was prepared using K_2_S_2_O_8_ and stored in the dark at room temperature for 12–16 h. The reagent test was then diluted with water to obtain an absorbance of 0.700 ± 0.02 at 734 nm. Ten microliters of each extract were added to 190 µL of the test reagent in a clear 96-well microplate and the absorbance was measured immediately at 734 nm. Trolox (0.1–0.5 mM) was used as standard and the results were expressed as milligrams of Trolox equivalents per gram of dry weight (mg_TE_/g_dw_).

#### 2.6.3. Ferric Reducing Antioxidant Power (FRAP)

The ability of the extracts to reduce ferric ions was measured following the procedure described by Yen and Chen [32]. FRAP methodology consists of the reduction of Fe (III) to Fe (II) in the presence of an antioxidant. Plant extract (100 μL) was mixed with K_3_[Fe (CN)_6_] solution (1%) (250 μL) and potassium phosphate buffer (200 mM, pH 6.6) (250 μL). The mixture was incubated for 20 min at 50 °C and after the addition of 10% TCA (250 μL), it was centrifuged for 10 min. The obtained supernatant (400 μL) was mixed with the same amount of water and 80 μL of 0.1% FeCl_3_. The absorbance was read at 700 nm to determine the reducing activity. Ascorbic acid was used as standard (0.0625–0.5 mM) and the results were expressed as milligrams of ascorbic acid equivalents per gram of dry weight (mg_AAE_/g_dw_).

#### 2.6.4. Oxygen Radical Absorbance Capacity (ORAC) Assay

Oxygen radical absorbance capacity (ORAC) assay was performed based on the method described by Gillespie et al. [33]. Twenty-five microliters of plant extract were mixed with fluorescein solution (0.2 μM). The reaction mixture was incubated for 10 min at 37 °C followed by reaction initiation with 150 mM AAPH (25 μL). Fluorescence was read every 5 min, for 90 min, up to value zero at 485 nm excitation and 530 nm emission. The results were calculated as ORAC values using the differences of areas under fluorescein decay curve between the blank without plant extract and the sample. Trolox (6.25–50 μM) was used as standard and the results were expressed as milligrams of Trolox equivalents per gram of dry weight (mg_TE_/g_dw_).

### 2.7. Enzyme Inhibitory Activities

#### 2.7.1. Acetylcholinesterase (AChE) and Butyrylcholinesterase (BChE) Inhibitions

Based on Ellman’s method [34], the inhibition of AChE and BChE was carried out using a 96-well microplate reader (Tecan Infinite M200). One hundred and twenty-five microliters of 3 mM DTNB, 50 µL of 100 mM phosphate buffer (pH 8.0), 25 µL of 15 mM substrate (ATCI or BTCI) and extracts were mixed in the wells of the microplate. Twenty-five microliters of AChE or BChE were added and after 5 min the absorbance was measured at 405 nm. Galanthamine was used as standard, and the results were expressed as milligrams of galanthamine equivalents per gram of dry weight (mg_GE_/g_dw_).

#### 2.7.2. Tyrosinase (Tyr) Inhibition

Inhibition of Tyr was determined using L-DOPA as substrate [35]. The assay was conducted in a 96-well microplate, where 40 µL of extract were mixed with 40 µL of tyrosinase solution and 80 µL of phosphate buffer. Forty microliters of L-DOPA were added after an incubation period of 10 min at room temperature. After another equal incubation time, the absorbance was measured at 475 nm. Kojic acid was used as standard, and the results were expressed as milligrams of kojic acid equivalents per gram of dry weight (mg_KAE_/g_dw_). 

### 2.8. Statistical Analysis

All tests were carried out in triplicates and data represent mean ± standard error for the total number of experimental results. Data were analyzed by one-way analysis of variance (ANOVA), and Duncan’s new multiple range test (*p* < 0.05), and correlations were calculated using Pearson’s test. Statistical analyses were carried out using IBM SPSS Statistics for Windows, Version 26.0. Armonk, NY: IBM Corp. Data were auto-scaled and a principal component analysis (PCA) was performed using the statistical software SOLO v. 8.6 (Eigenvector Research Inc., Manson, WA, USA).

## 3. Results and Discussion

### 3.1. Optimization of Extraction Conditions

**Figure 1:**
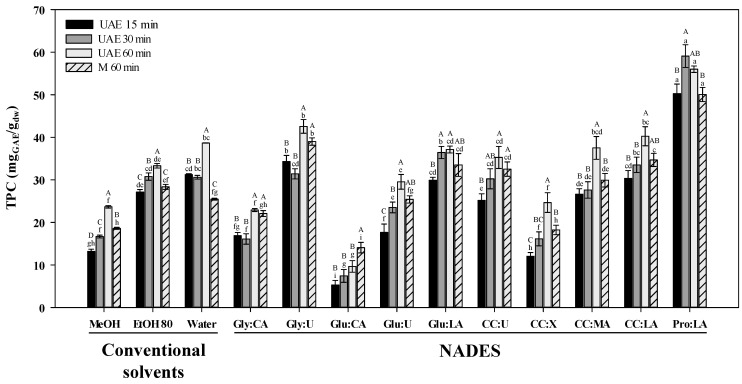
Effect of extraction technique (M: maceration and UAE: ultrasound-assisted extraction), extraction period (15, 30 and 60 min) and solvent (conventional and natural deep eutectic solvents, NADES) on total phenolic contents (TPC) of *Lavandula pedunculata* subsp. *lusitanica* extracts. The corresponding to the solvent’s abbreviations can be consulted in Table 1. Values are expressed as mean ± SE (*n* = 3). Different letters in each series indicate significant differences (*p* < 0.05), (Duncan’s new multiple range test). Uppercase letters indicate significant differences (*p* < 0.05) between the four extraction conditions (M60, UAE 60, 30, 15) while lowercase letter denotes significant differences (*p* < 0.05) between solvents.

In order to compare the efficiency of UAE to maceration, the extractions were performed with control of temperature and using the same solvents (conventional and NADES) for 60 min, since some authors reached the maximum recoveries of phenolics with this extraction period [23]. The desirable extraction temperature ranges from 25 °C to about 60 °C [15]. In this study 50 °C was used as extraction temperature. The TPC of the different extracts, determined by F-C method, were compared to assess the best extraction conditions (Figure 1). UAE was more efficient (TPC from 22.90–56.00 mg_GAE_g_dw_) than maceration (18.22–50.05 mg_GAE_/g_dw_) for all solvents, excepting Glu:CA, in which maceration extracted a larger amount of phenolics (14.07 ± 1.24 vs. 9.64 ± 1.36 mg_GAE_/g_dw_). Similar results were obtained by other authors [36,37]. In addition, Jeong et al. [38] and Nam et al. [39] compared four extraction methods (stirring, heating, heating with stirring, and UAE) using DES to extract monoterpenes and phenolic compounds from *Mentha piperita* L. and flavonoids from *Flos sophorae*, respectively, and obtained a greater extraction efficiency with UAE. 

After proving that UAE was better than maceration, the influence of three extraction periods—15, 30 and 60 min (UAE 15, UAE 30 and UAE 60)—was also tested to find out if a shorter period was sufficient for a good extraction of bioactive compounds. In agreement with Charpe and Rathod [40], the extraction efficiency increased with extraction time rise (Figure 1). In general, the highest TPC values were obtained by UAE 60, excepting for Pro:LA mixture with TPC values decreasing after 30 min. Although no significant differences were observed between 15 and 30 min, the extraction efficiency was higher at 30 min. In water and glycerol-based NADES, UAE 15 showed a slight increase. In the case of NADES, the higher TPCs for UAE 15 could be explained by phenolic compounds interaction with NADES to form polymers for longer extraction periods, or the extraction stability might have been affected by solvents polarity [41]. Similar results were obtained by Zhou et al. [41], which tested 10, 20, 30, 40, and 50 min to extract phenolic compounds from *Morus alba* L. leaves using UAE and DES, and obtained a higher extraction yield at 30 min. Bajkacz and Adamek [23] tested extraction times ranged from 40 to 120 min to extract isoflavones from soy products and recoveries reached a maximum at 60 min, and did not increase further as the extraction proceeded. 

Overall, UAE 60 was the most adequate to extract phenolic compounds from *L. pedunculata* and was further used to evaluate the effect of extraction solvent on phenolic profile and bioactivity of the extracts.

### 3.2. Solvents Effect on Phenolic Compounds (F-C method and HPLC-HRMS), Antioxidant Activity and Enzyme Inhibitory Capacities

Ten NADES were prepared by heating and stirring methods [21] using four groups of HBAs—glycerol, glucose, choline chloride and proline—in combination with three groups of HBDs—three organic acids (citric, lactic and malic acids), one polyalcohol (xylitol), and urea (Table 1). The NADES components selected to be tested in this work were approved as safe by FDA, as can be attested by their CRFs (Code of Federal Regulations) [42]. In addition, conventional solvents (water, EtOH 80 and MeOH) were used for comparison purposes. 

#### 3.2.1. Total and Individual Phenolic Contents

A total of twenty-four phenolic compounds were identified, seventeen in quantifiable amounts (Supplementary Figure S1 and Table S2). Salvianolic acid A isomer II was detected in all the extracts but below the limit of detection (not present in Table 2). The identification of these compounds led to their distribution into five structurally related classes/groups, i.e., hydroxycinnamic and hydroxybenzoic acids (phenolic acids), flavones and flavanones (flavonoids), and a coumarin derivative (Table 2 and Supplementary Table S1). 

The most abundant compounds in all the extracts were the hydroxycinnamic acids, in agreement with the literature available for other *Lavandula* species [28,43,44]. On the other hand, Contreras et al. [27] showed that in hydromethanolic extracts of *L. stoechas* and *L. dentata* the hydroxycinnamic acids made up the largest class in both extracts but flavones were the most abundant class.

In general, rosmarinic acid was the most abundant compound found in the extracts, followed by ferulic acid and salvianolic acid B (Table 2). These results are in accordance with results previously obtained for other *Lavandula* species [28,43,45]. Lopes et al. [5] identified thirteen compounds in *L. pedunculata* (Mill.) Cav., being salvianolic acid B and rosmarinic acid the major compounds. Similarly, Costa et al. [4] also reported rosmarinic acid as the most abundant phenolic in *L. pedunculata* subsp. *lusitanica* in all studied extracts (ethanolic—50 and 100%—extracts and water infusion), excepting in the water extract at room temperature, in which 4-O-caffeoylquinic acid was superior. Although rosmarinic acid was the most abundant phenolic compound in most extracts, ferulic acid was found in predominant amounts in water, Gly:U and Glu:U extracts, as previously observed in *L. vera* [46] and *L. angustifolia* [44].

Although no significant differences were observed among the conventional solvents MeOH and EtOH 80 and the choline chloride (CC) and lactic acid (LA)-based NADES in the extraction of rosmarinic acid, the green solvents, namely CC:U and CC:LA, were more efficient to extract ferulic acid than MeOH and EtOH 80. Similar results were obtained by Xie et al. [47] being CC:U the best solvent for extracting ferulic acid (*p* < 0.05) compared to other NADES and conventional solvents. Other authors reported CC:U (1:2) as an excellent solvent for the extraction of compounds from other genus, namely rutin from *Sophora japonica* [48], 3,4-dicaffeoylquinic acid from *Lonicerae japonicae* Flos [49] and tyrosol from extra-virgin olive oil [50]. In addition to ferulic acid, in our study, CC:U extracted the highest amounts of fertaric acid (*p* < 0.05). 

Overall, no significant differences were observed among the conventional solvents EtOH 80 and water and NADES containing LA or CC in their formulation, for the salvianolic acid B extraction (Table 2), showing Pro:LA and CC:LA significant better results than MeOH. Our results are in agreement with those by He et al. [51] that extracted the largest amount of salvianolic acid B from *Salvia miltiorrhiza* with Pro:LA (1:1) after testing UAE combined with sixteen different NADES and two conventional solvents (water and methanol). It should also be noted that Pro:LA also stood out in TPC (Figure 1), showing a significant highest content for all extraction conditions tested (50.05–59.09 mg_GAE_/g_dw_).

NADES, including the CC as HBA or LA as HBD, showed a comparable potential to extract (total) phenolic acids (Table 2) as MeOH and EtOH 80. Regarding flavonoids, just overcome by water, the Pro:LA, CC:LA, Glu:LA and CC:X NADES and the convectional solvents MeOH and EtOH 80 were the second-best extractants. Moreover, as for phenolic acids, LA-based NADES showed a good efficiency to extract flavonoids, indicating that the polarity of these green solvents and their hydrogen bonding interactions with these compounds appears to be very important. 

Analyzing the TPCs obtained by UAE at the longer extraction period (Figure 1), NADES with organic acids as the HBDs (with exception for citric acid) yielded higher extraction efficiencies than MeOH. This might be due to the lower viscosity of LA containing solvents [with one carboxyl group (COOH)] compared to solvents formed by citric acid (CA) (with three carboxyl groups); the increase in intermolecular forces in this last molecule, provide a higher viscosity and therefore impairing the extraction of phenolics [52]. However, the presence of several carboxyl or hydroxyl groups should not be overlooked because allows more hydrogen bonds to be formed, increasing the stability of the liquids [53]. This can be supported by comparing HPLC results of extracts of LA and malic acid (MA) both in combination with CC. Although MA presents one more carboxyl group, when in NADES the amount of LA was increased in relation to MA, and the changes in the extraction behavior are probably due to the increase in hydroxyl groups. In fact, CC:LA was more effective in extracting (total) flavonoids and some phenolic acids (vanillic, caffeic and ferulic acids and feruloyl hexose) than CC:MA. Cui et al. [54] shown that the increase in HBD proportion reduces the viscosity and surface tension of the solvent, promoting the diffusion and enhancing the mass transfer, which can be another reason (despite being different acids) to explain the higher extraction capacity of CC:LA (1:2) compared to CC:MA (1:1) in most of the conditions tested (M60 min and UAE 30 min). On the other hand, the poorest recovery of phenolic compounds (phenolic acids and flavonoids), analyzed by HPLC and TPC, were obtained with Glu:CA which could be attributable to the high viscosity of CA-based NADES (with three carboxyl groups), which hindered the efficiency as extraction solvents due to their low mass transport [16]. These results are in agreement with those by Nam et al. [39], showing that Glu:CA (1:1) was the worst green solvent for the extraction of flavonoids (quercetin, kaempferol and isorhamnetin) from *Flos sophorae* and TPCs from eucalyptus leaves [55]. Different results were observed by Liu et al. [52] that extracted the highest amounts of curcuminoids with Glu:CA (1:1). 

Overall, HPLC data suggests that NADES, including CC or LA in their composition, with special emphasis for Pro:LA and CC:U, proved to be equally efficient (or better in some cases) to extract phenolic compounds when compared to conventional solvents (Table 2). In this sense, the methodology proposed by means of NADES for the extraction of phenolics from *L. pedunculata* can be considered as a greener alternative in comparison with organic solvents used until now for the same purpose such as ethanol/water (80:20 *v/v*) [5], n-hexane, dichloromethane, ethyl acetate and methanol [6] and ethanol/water (50:50 *v/v*) [4].

#### 3.2.2. Antioxidant Activity

Plant extracts are multicomponent mixtures exceedingly complex and for this reason it is important to evaluate the antioxidant capacity by more than one assay. In this work it was evaluated by using four different chemical assays with two distinct mechanisms, one single electron transfer-based method–FRAP, one atom hydrogen transfer-based method–ORAC, and two mixed methods using hydrogen-atom transfer and single-electron transfer–DPPH and ABTS [26,56].

In all the assays the conventional solvents EtOH 80 and water displayed better results than MeOH. In ORAC and FRAP, EtOH 80 proved to be a better extractant than water (*p* < 0.05), whereas in DPPH, no significant differences were obtained between those solvents. Conversely, in ABTS, water showed higher antioxidant activity values than hydroalcoholic solution (54.66 ± 1.09 vs. 46.31 ± 0.85 mg_TE_/g_dw_). Comparing conventional solvents, different results are found in literature. In *L. pedunculata* subsp. *lusitanica* (Chaytor) Franco, Costa et al. [4] observed that water extracts exhibited higher activity than water:ethanol (1:1) in ORAC assay. On the other hand, Lopes et al. [5] reported that hydroethanolic (80:20, *v/v*) extracts from *L. pedunculata* (Mill.) Cav. had higher DPPH scavenging activity and reducing power (FRAP) than water extracts.

**Figure 2:**
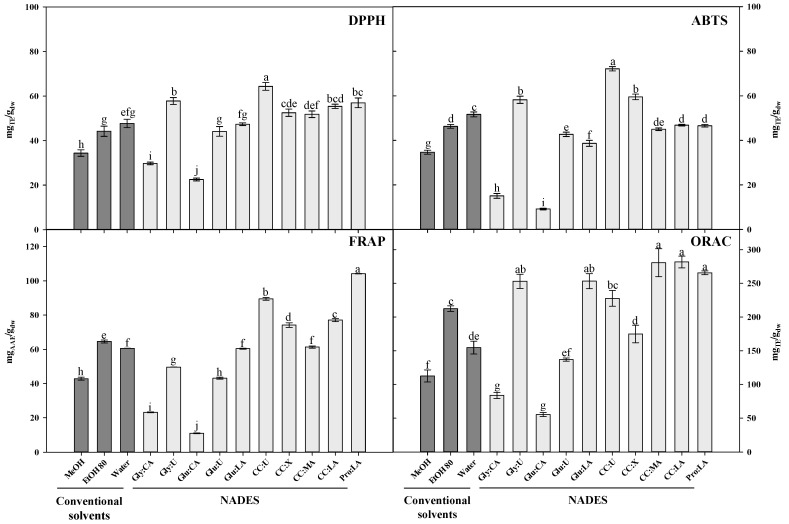
Effect of conventional and natural deep eutectic solvents on antioxidant capacity, determined by 2,2-diphenyl-1-picrylhydrazyl (DPPH), 2,2′-azino-bis(3-ethylbenzothiazoline-6-sulfonic acid) (ABTS), ferric reducing antioxidant power (FRAP) and oxygen radical absorbance capacity (ORAC) assays, of extracts from *Lavandula pedunculata* subsp. *lusitanica* obtained by ultrasound-assisted extraction for 60 min. The corresponding to the solvent’s abbreviations can be consulted in Table 1. Values are expressed as mean ± SE (*n* = 3). Different letters in each graph bars indicate significant differences (*p* < 0.05, Duncan’s new multiple range test).

Overall, the highest free radical scavenging capacity (DPPH^•^ and ABTS^•+^) was obtained in CC:U extracts (Figure 2). The ABTS radical scavenging ability observed with this solvent (72.13 ± 0.97 mg_TE_/g_dw_) was around twice higher than that obtained with MeOH (34.67 ± 0.90 mg_TE_/g_dw_) and EtOH 80 (46.31 ± 0.85 mg_TE_/g_dw_), and almost eight times greater than that of Glu:CA (9.17 ± 0.38 mg_TE_/g_dw_), which was considered the worst solvent in this study. Indeed, Glu:CA was the solvent with the lower antioxidant activity in all assays, which is consistent with total phenolic content from HPLC (Table 2) and F-C method (Figure 1), indicating that phenolic compounds are important contributors to the antioxidant properties in *L. pedunculata*. These results are in agreement with Gullón et al. [55], which showed a substantially lower antioxidant activity in extracts from *Eucalyptus globulus* leaves using Glu:CA in the same ratio used in this work. 

In addition to DPPH and ABTS scavenging capacity, CC:U extract also exhibited a good ability to reduce ferric ions, 89.50 ± 0.81 mg_AAE_/g_dw_, which corresponds to the second-best solvent in FRAP assay. Pro:LA was the extract with the highest FRAP value (104.19 ± 0.10 mg_AAE_/g_dw_), one of the extracts with highest ORAC value (265.56 ± 2.83 mg_TE_/g_dw_) and the second-best result in DPPH assay (56.96 ± 2.18 mg_TE_/g_dw_). Good results of DPPH scavenging effect (%) were also obtained with Pro:LA (1:1) by Rajan and Ramesh [57] and He et al. [51], for *Zingiber officinale* (89.33% at 3.33 mg/mL) and *Salvia miltiorrhiza* (≈ 87% at 10 mg/mL), respectively. 

All CC-based NADES provided best FRAP results than conventional solvents, except for CC:MA that, despite being better than MeOH, had similar results as EtOH 80. In ORAC assay, CC:MA and CC:LA also provided higher results (Figure 2), which are not significantly different from those of Pro:LA, Gly:U and Glu:LA extracts. Radošević et al. [58] tested the antioxidant activity by ORAC of five grape skin extracts obtained with NADES and observed the highest value with CC:MA (1:1). 

The small differences among antioxidant results can be explained by the different mechanisms of action involved in the different methods. Overall, the solvents that showed the highest activities were Pro:LA and CC:U. On the other hand, MeOH and EtOH 80 proved to be better that CA-based NADES. These results reinforce the TPC data obtained by F-C and HPLC methods. 

#### 3.2.3. Enzyme Inhibitory Capacity 

**Figure 3:**
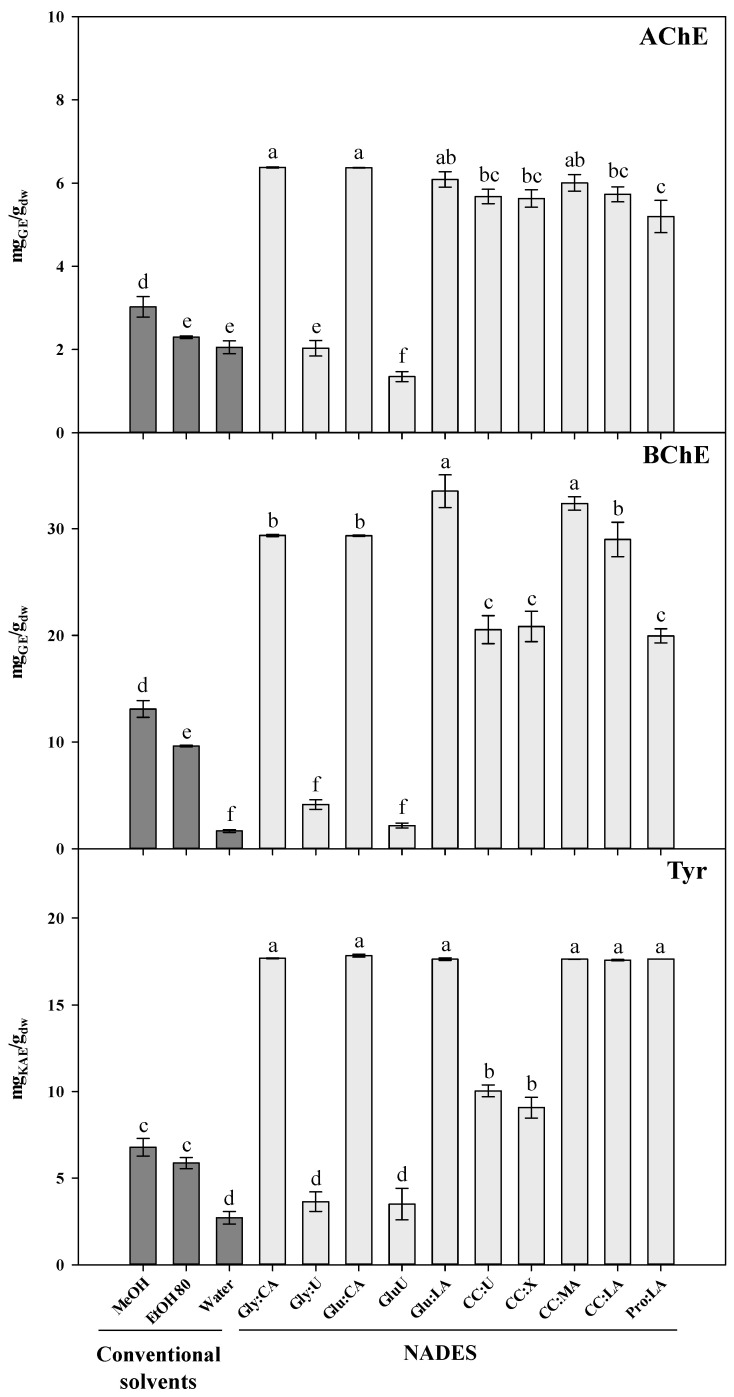
Effect of conventional and natural deep eutectic solvents on acetylcholinesterase (AChE), butyrylcholinesterase (BChE) and tyrosinase (Tyr) inhibitory activities of extracts from *Lavandula pedunculata* subsp. *lusitanica* obtained by ultrasound-assisted extraction for 60 min. The corresponding to the solvent’s abbreviations can be consulted in Table 1. Values are expressed as mean ± SE (*n* = 3). Different letters in each graph bars indicate significant differences (*p* < 0.05, Duncan’s new multiple range test).

The potential of NADES-based *L. pedunculata* extracts to inhibit three enzymes—AChE, BChE and Tyr—involved in neurodegenerative diseases were investigated and the results depicted in Figure 3. To the best of our knowledge, no published studies regarding AChE and BChE inhibition capacity of NADES itself or NADES extracts were found in the literature. Only one study was found about the Tyr inhibition capacity of *Ixora javanica* flower extracts obtained with various DES [59]. 

In general, methanolic extracts showed the highest inhibition of cholinesterases followed by EtOH 80 and water extracts (Figure 3). In the case of AChE inhibition, no significant differences were found between EtOH 80 and water extracts. Conversely, when Costa et al. [4] evaluated the AChE and BChE inhibitory activities of polar extracts [water, ethanol:water (50:50, *v/v*), and ethanol] from the same species found that ethanol showed the highest inhibition capacity for AChE, but no significant differences between solvents were obtained for BChE. 

Our results show that all organic acids-based extracts (with strong acid pH) had the highest capacity to inhibit AChE and BChE (5.73–6.38 mg_GE_/g_dw_ and 28.99–33.51 mg_GE_/g_dw_, respectively), except Pro:LA that presented a slightly lower inhibition for BChE. This evidence can be possibly explained because Pro:LA has a less acidic pH and closer to the optimal enzyme pH (7.4 for AChE) [60]. According to Çakıroğlu et al. [61] the activity of AChE decreased substantially from the optima pH with the pH decrement, which shows the acid labile nature of AChE. In this way, extracts from NADES elaborated with organic acids itself seem to contribute for a significant part of this inhibition. No studies were found in the literature regarding cholinesterases inhibition and NADES as extractants. 

Tyrosinase is a copper-containing enzyme, also known as polyphenol oxidase (PPO). Besides their inhibitors being attractive as anti-neuromelanin agents in Parkinson’s and Huntington’s diseases, the study of Tyr inhibition is also an active field of research in other industries, namely cosmetics and dermatological (as depigmentation agents), and food and agricultural (as antibrowning compounds). Several medicinal plants are considered Tyr inhibitors mainly due to flavonoids and stilbenes [62,63,64].

To our best knowledge, this is the first time that Tyr inhibition capacity of *L. pedunculata* has been evaluated. Only two studies were found in the literature related to this enzyme and *Lavandula* genus. Sariri and Seifzadeh [65] and Lee and colleagues [66] investigated the capacity of extracts from other lavender species as Tyr inhibitors and both showed Tyr-inhibitory activity, but with higher IC_50_ values than kojic acid. In this way, it was also expectable that *L. pedunculata* was not a potent Tyr inhibitor, which is supported by the lowest result obtained in this work by the water extract (2.71 ± 0.37 mg_KAE_/g_dw_). However, unlike the other solvents and similarly to cholinesterases inhibition, all organic acids-based extracts had the higher inhibition with values around 18 mg_KAE_/g_dw_ (Figure 3). Oktaviyanti et al. [59] used various DES composed with organic acids as extractants of bioactive compounds from *I. javanica*, such as CC:LA (1:2) and CC:MA (1:1), obtaining inhibition of 65% and 72% at 50 mg/mL, respectively. Since extracts of conventional solvents presented lower potential to inhibit Tyr (range from 2.71 to 6.78 mg_KAE_/g_dw_) than organic acids-based extracts, it is possible that organic acids (lactic, malic, and citric acids) also contribute to the activity of the extract. This evidence is in agreement with Moon et al. [67] that reviewed chemical methods developed to inhibit the activity of PPOs and reported that the optimal enzyme’s activity is at pH 5–7 and its inhibition occurs below pH 3.0. Still according to them, acidifying agents, such as citric acid and ascorbic acid can inactivate these enzymes by lowering the pH. Furthermore, citric acid is a copper-chelating agent, able to suppress PPO activity by binding to metal cofactors in the enzyme structure. Regarding lactic and malic acids, their inhibitory effects on enzymatic browning derive from their lowering pH or metal-chelating traits.

#### 3.2.4. Pearson’s Correlation between the Different Parameters (Phenolics, Antioxidant Capacities and Enzyme Inhibitory Activities) Evaluated and Principal Component Analysis (PCA)

In this study, a strong correlation was observed between antioxidant results and total phenolic contents by F-C method (*p* < 0.01). The phenolic contents by HPLC were strongly correlated with antioxidant results observed for FRAP (*p* < 0.01) and moderately correlated for DPPH, ABTS and ORAC (*p* < 0.05) (Table 3). The high correlations found between F-C assay results and the antioxidant results obtained by spectrophotometric methods, in relation to the analysis performed by HPLC, can be explained because the F-C method presents some limitations (e.g., poor specificity). This assay can be influenced by any other substances that can be oxidized by the Folin reagent producing an overestimation of the results. The phenolic profile obtained by HPLC is only strongly correlated with FRAP results, which can evidence again the different mechanisms of action involved in the assay. Furthermore, previous investigations in other species have shown that the presence of several potential antioxidant constituents (i.e.; fatty acids, sulfur-containing glucosides and chlorophylls) could be implicated in the neutralization of free radical damage [68]. Despite the influence of other compounds on the antioxidant activity [69], the high correlations between the phenolic content obtained by the chromatographic and spectrophotometric methods and antioxidant activities evaluated by different assays demonstrate that these compounds are the ones that mainly characterize the antioxidant potential of the *L. pedunculata* extracts. These results further suggest that the NADES tested act as good extractants of bioactive compounds from this plant.

Rosmarinic acid is a caffeic acid ester found in a variety of plants of the Lamiaceae family such as lavender that has shown several interesting biological activities, including antioxidant activity [26,44]. Although it is possible to observe a strong correlation between TPC and antioxidant activity and besides, rosmarinic acid is the most abundant compound in almost all extracts, in our study this compound did not show a strong correlation with antioxidant activity results, unlike ferulic acid, which revealed a correlation higher than 0.708 (*p* < 0.01) in all antioxidant tests (Table 3). Therefore, it is possible that ferulic acid may be one of the greatest contributors to the antioxidant potential of the *L. pedunculata* extracts observed in this work. This fact is very clear in Gly:U, since ferulic acid is the single abundant compound in the extract and its antioxidant activity was very evident, particularly in DPPH and ABTS assays (Table 2 and Figure 2). Furthermore, the radical scavenging capacity of ferulic acid has been previously demonstrated by some authors [70,71]. Although fertaric acid, feruloyl hexose, salvianolic acid A isomer I and luteolin-7-O-glucuronide are present in low amounts in the extracts in comparison to the major compounds, there is a strong correlation (*p* < 0.01) between these compounds and most of the antioxidant results, suggesting that these compounds might also contribute to the radical scavenging capacity displayed by the extracts. 

It has been demonstrated by many reports that phenolic compounds, specially flavonoids, are, in general, good inhibitors of Tyr [62] and cholinesterases [10,72,73]. However, despite the highest concentration of rosmarinic acid in the extracts (Table 2) and its recognized role as Tyr inhibitor [74,75], no correlation was observed between this compound and enzymes inhibitory activities (Table 3). In fact, the phenolic compounds that appear to have a contribution to the inhibition of Tyr enzyme are the luteolin glycosides tentatively identified in this work, with a strong correlation (*p* < 0.01) in the case of luteolin-7-O-glucoside. This flavone revealed a noncompetitive and moderated Tyr inhibition in previous studies [76,77]. Luteolin-7-O-glucoside showed a moderated correlation with AChE and BChE in the present study and has been reported as AChE and BChE inhibitor previously [78,79]. Luteolin-7-O-glucuronide showed a moderated correlation (*p* < 0.05) with the three enzymes. Although no studies related with the inhibition of the three mentioned enzymes by this flavonoid were found in the literature, luteolin-7-O-glucuronide inhibits the enzyme matrix metalloproteinases (MMP) responsible for the collagen and other protein degradation in extracellular matrix [80]. These results also indicate that the main influence of the inhibition to these enzymes comes from these two flavones, discarding the influence of other effects that the evaluated green solvents might have.

**Figure 4:**
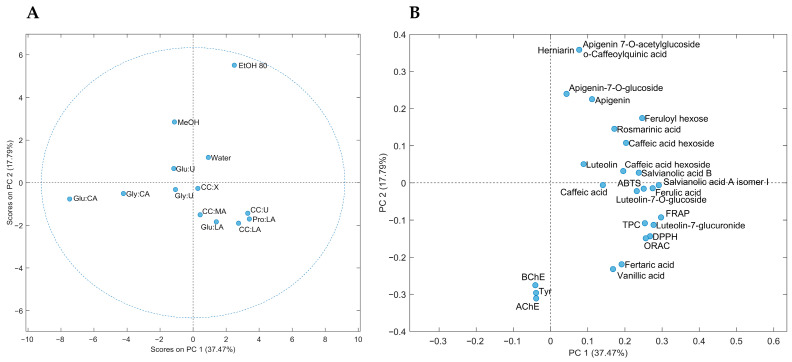
Score plot (**A**) and loading plot (**B**) of principal component analysis (PCA) of extracts from *Lavandula pedunculata* subsp. *lusitanica* using NADES and conventional solvents.

Principal component analysis (PCA) (Figure 4) was used to explore the similarities or differences among different *Lavandula* extracts obtained using convectional and green solvents, and based on the parameters of study, the individual and total phenolic content, and the antioxidant and enzyme (AChE, BChE and Tyr) inhibitory activities. Each point on the score plot (Figure 4a) represents extracts from each tested solvent, while each point on the loading plot (Figure 4b) represents the contribution of each variable/parameter to the score. The first two principal components (PC) accounted for 55.26% of the total variation in the dataset, where the first principal component (PC1) explained 37.47% of the data variability in the dataset, whereas the second principal component (PC2) explained 17.79%. 

The score plot of the first two principal components (Figure 4a) showed a clear grouping of the extracts by type of solvent. In general, it is possible to observe a clear separation between conventional solvents (positive PC2 values, first and second quadrants) and NADES-based (mainly negative PC2 values, third and fourth quadrants) extracts. The results suggest that each type (convectional or green) of solvent presents a different ability to extract different classes of compounds or relative concentrations. EtOH 80 extract, located in the first quadrant and with the highest contribution to PC2, presented the highest distinction among extracts. It is the best solvent to extract apigenin and derivatives, as well as O-caffeoylquinic acid and herniarin (Figure 4b). Regarding extracts using NADES (composed by 30% water) and water, both types of solvents presented more extraction similarities than those of MeOH and EtOH 80. Water extract, positioned in the first quadrant and with low contribution to both principal components (PC1 and PC2), is mainly characterized by the flavonoid luteolin. On the other hand, those NADES extracts positioned in the fourth quadrant and containing CC as HBA (mainly CC:U and CC:LA), and LA as HBD (mainly Pro:LA and CC:LA), presented similarities and are confirmed as good extractants of compounds such as phenolic acids with different polarities (caffeic, vanillic, fertaric, ferulic, rosmarinic and salvianolic acids; and derivatives, feruloyl hexose and caffeic acid hexosides), and the flavonoid luteolin and its derivatives. These CC and LA extracts also exhibited the highest antioxidant activities. Finally, CA-based NADES (mainly Glu:CA) extracts, located in the third quadrant, are defined by the lowest antioxidant activities and phenolic contents. 

## 4. Conclusions

In this study an environmentally friendly, economic, and efficient approach based on NADES in combination with UAE was applied for the extraction of phenolic compounds from *Lavandula pedunculata* subsp. *lusitanica*. UAE for 60 min was the best extraction condition tested, and Pro:LA and CC:U were the green solvents that extracted the highest total phenolic contents (by HPLC and F-C methods) and showed the maximum antioxidant activity. Strong extraction efficiency of the main phenolics identified in *L. pendunculata* subsp. *lusitanica* extracts such as the phenolic acids rosmarinic, ferulic and salvianolic acid B and the flavonoids luteolin 7-glucuronide, luteolin and apigenin were obtained with NADES (mainly CC and LA-based solvents). The advanced method described showed good potential to be considered as a new green technique for lavender bio-active compounds extraction with higher phenolic contents and potential application in pharmaceutical and food industries. Further studies based on toxicology limits, as well as about the nutritional and potential health benefits (clinical studies) are required to validate these extracts as potential food matrices with different biological properties [e.g., enzyme (tyrosinase and cholinesterases) inhibitors]. In parallel, since NADES can be also used in food matrices, it may be also interesting to evaluate the effect of storage time with empirical models to describe the degradation reactions of these matrices.

## Figures and Tables

**Table 1:** Composition of natural deep eutectic solvents (NADES) used in this study and details concerning the synthesis thereof.

Abbreviations	Component 1 (HBA)	Component 2 (HBD)	Molar Ratio	Appearance
Gly:CA	Glycerol	Citric acid	2:1	Transparent light-yellow semi viscous liquid
Gly:U	Glycerol	Urea	1:1	Transparent colourless liquid
Glu:CA	Glucose	Citric Acid	1:1	Faintly yellow viscous liquid
Glu:U	Glucose	Urea	1:2	Transparent colourless semi viscous liquid
Glu:LA	Glucose	Lactic Acid	1:5	Transparent colourless liquid
CC:U	Choline Chloride	Urea	1:2	Transparent colourless liquid
CC:X	Choline Chloride	Xylitol	2:1	Transparent colourless liquid
CC:MA	Choline Chloride	Malic Acid	1:1	Transparent colourless liquid
CC:LA	Choline Chloride	Lactic Acid	1:2	Transparent colourless liquid
Pro:LA	Proline	Lactic Acid	1:1	Transparent colourless liquid

**Table 2:** Qualitative and quantitative (µg/g of extract, mean) analysis by high performance liquid chromatography-high resolution mass spectrometry (HPLC-HRMS) of phenolic profile from *Lavandula pedunculata* subsp. *lusitanica* extracts obtained by ultrasound-assisted extraction for 60 min.

Compound	Conventional Solvents			NADES									
	MeOH	EtOH 80	Water	Gly:CA	Gly:U	Glu:CA	Glu:U	Glu:LA	CC:U	CC:X	CC:MA	CC:LA	Pro:LA
O-Caffeoylquinic acid	<LOQ	68.41	<LOQ	n.d.	n.d.	n.d.	n.d.	n.d.	n.d.	n.d.	n.d.	n.d.	n.d.
Gallic acid	<LOD	n.d.	<LOD	n.d.	n.d.	n.d.	<LOD	n.d.	n.d.	n.d.	n.d.	n.d.	n.d.
Vanillic acid	<LOQ	<LOQ	<LOQ	<LOQ	68.03 ^b^	<LOQ	78.69 ^b^	86.98 ^b^	91.35 ^b^	<LOQ	<LOQ	97.55 ^b^	179.1 ^a^
Caffeic acid hexoside	79.90 ^bc^	67.76 ^bcd^	62.22 ^cd^	53.25 ^d^	<LOQ	<LOQ	106.1 ^a^	77.97 ^bc^	86.24 ^b^	64.47 ^cd^	68.43 ^bcd^	81.32 ^bc^	72.92 ^bcd^
Caffeic acid hexoside	112.8 ^a^	93.26 ^abc^	72.44 ^cd^	60.23 ^d^	<LOQ	<LOQ	100.1 ^ab^	89.62 ^bc^	100.5 ^ab^	72.22 ^cd^	79.22 ^bcd^	81.64 ^bcd^	86.19 ^bc^
Chlorogenic acid (5-O-caffeoylquinic acid)	<LOD	<LOD	<LOD	<LOD	<LOD	n.d.	<LOD	n.d.	<LOD	n.d.	<LOD	n.d.	n.d.
Fertaric acid	53.15 ^g^	163.1 ^f^	278.8 ^bc^	174.6 ^ef^	283.5 ^b^	91.70 ^g^	229.4 ^bcde^	277.8 ^bc^	341.7 ^a^	208.8 ^ef^	213.5 ^def^	270.2 ^bcd^	223.7 ^cde^
Caffeic acid	81.50 ^cd^	75.14 ^d^	427.4 ^a^	103.4 ^cd^	<LOD	<LOQ	90.72 ^cd^	160.0 ^c^	248.3 ^b^	76.68 ^cd^	<LOQ	157.3 ^cd^	137.6 ^cd^
Feruloyl hexose	259.8 ^a^	233.5 ^b^	201.2 ^c^	97.46 ^g^	195.8 ^cd^	56.95 ^h^	140.5 ^f^	197.7 ^c^	212.0 ^bc^	163.6 ^ef^	170.4 ^de^	208.7 ^bc^	184.9 ^cde^
Ferulic acid	1846 ^e^	3079 ^c^	3657 ^ab^	1427 ^f^	3020 ^c^	546.9 ^g^	2868 ^cd^	3105 ^c^	3774 ^a^	2557 ^d^	2979 ^c^	3417 ^b^	2585 ^d^
Rosmarinic acid	7224 ^a^	6882 ^a^	2416 ^cde^	2973 ^bcde^	59.66 ^e^	1620 ^de^	1556 ^de^	4252 ^abcd^	5239 ^abc^	5828 ^ab^	4181 ^abcd^	4375 ^abcd^	6089 ^ab^
Salvianolic acid A isomer I	57.46 ^c^	121.2 ^ab^	95.52 ^bc^	58.66 ^c^	46.13 ^c^	<LOQ	81.72 ^bc^	90.11 ^bc^	158.8 ^a^	104.7 ^abc^	87.88 ^bc^	101.5 ^abc^	132.2 ^ab^
Salvianolic acid I	<LOD	<LOQ	<LOQ	<LOD	<LOD	<LOD	<LOD	<LOQ	<LOD	<LOQ	<LOQ	<LOQ	<LOQ
Salvianolic acid B	930.9 ^bcd^	2322 ^a^	1414 ^abc^	701.2 ^bcd^	<LOD	342.3 ^cd^	233.7 ^d^	1710 ^ab^	1220 ^abcd^	1472 ^abc^	1527 ^ab^	2144 ^a^	2277 ^a^
Salvianolic acid A isomer III	<LOD	<LOD	<LOD	<LOD	<LOD	<LOD	<LOD	<LOD	<LOD	<LOD	<LOD	<LOD	<LOD
**Total Phenolic Acids**	10646 ^ab^	13106 ^a^	8624 ^bc^	5649 ^cd^	3673 ^d^	2658 ^d^	5485 ^cd^	10048 ^ab^	11473 ^ab^	10549 ^ab^	9307 ^abc^	10934 ^ab^	11967 ^ab^
Luteolin-7-O-glucuronide	84.32 ^gh^	216.6 ^bcd^	97.82 ^fgh^	66.94 ^h^	145.7 ^ef^	<LOD	121.3 ^efg^	265.7 ^ab^	209.2 ^cd^	170.4 ^de^	203.7 ^cd^	291.9 ^a^	231.4 ^bc^
Luteolin-7-O-glucoside	58.11 ^cde^	71.07 ^bc^	<LOD	<LOQ	25.97 ^f^	<LOD	<LOQ	64.38 ^cd^	45.98 ^de^	44.07 ^e^	52.84 ^cde^	91.15 ^a^	83.54 ^ab^
Apigenin-7-O-glucoside	98.05 ^a^	73.68 ^a^	<LOD	31.59 ^b^	95.37 ^a^	<LOQ	44.44 ^b^	25.20 ^b^	32.84 ^b^	30.31 ^b^	24.74 ^b^	28.22 ^b^	37.23 ^b^
Apigenin-7-O-acetylglucoside	<LOD	30.67	<LOD	<LOQ	<LOD	<LOD	<LOQ	<LOQ	<LOQ	<LOQ	<LOQ	<LOQ	<LOQ
Luteolin	154.1 ^c^	78.26 ^ef^	632.8 ^a^	90.82 ^de^	<LOD	32.22 ^f^	<LOD	94.14 ^de^	114.4 ^cde^	171.1 ^bc^	58.78 ^ef^	138.8 ^cd^	215.3 ^b^
Naringenin	<LOQ	<LOQ	<LOD	<LOD	<LOD	<LOD	<LOD	<LOD	<LOD	<LOD	<LOD	<LOD	<LOD
Apigenin	242.8 ^a^	208.4 ^ab^	267.3 ^a^	126.1 ^bcd^	<LOD	63.78 ^d^	55.70 ^d^	131.9 ^bcd^	92.54 ^cd^	129.0 ^bcd^	111.6 ^bcd^	140.3 ^bcd^	175.1 ^abc^
**Total Flavonoids**	637.4 ^bcd^	678.6 ^bc^	998.0 ^a^	315.4 ^efg^	267.0 ^fgh^	96.00 ^h^	221.4 ^gh^	581.3 ^bcd^	495.0 ^cde^	544.9 ^bcd^	451.6 ^def^	690.4 ^bc^	742.6 ^b^
Herniarin	<LOQ	43.96 ± 6.05	n.d.	n.d.	n.d.	n.d.	n.d.	n.d.	n.d.	<LOQ	<LOQ	n.d.	<LOQ
**Total Phenolic Compounds**	11283 ^a^	13828 ^a^	9623 ^ab^	5965 ^bc^	3940 ^c^	2754 ^c^	5707 ^bc^	10629 ^a^	11968 ^a^	11094 ^a^	9759 ^ab^	11625 ^a^	12709 ^a^

Notes: n.d.—not detected; LOD—limit of detection; LOQ—limit of quantification. The results were analyzed using one-way analysis of variance (ANOVA) followed by Duncan’s new multiple range test. Different letters (a–h) in each row and for each phenolic compound mean significant differences (*p* < 0.05) among extracts.

**Table 3:** Pearson’s correlation coefficients between antioxidant activity measured by the different assays (DPPH, FRAP, ABTS, and ORAC), enzyme inhibitory activities (AChE, BChE, and Tyr), total phenolic contents measured by F-C and HPLC, and individual phenolic compounds.

Individual Phenolic Compounds		Antioxidant Activity				Enzyme Inhibitory Activity		
		DPPH	FRAP	ABTS	ORAC	AChE	BChE	Tyr
Fertaric acid		0.799 **	0.561 **	0.688 **	0.605 **	−0.010	−0.104	−0.087
Ferulic acid		0.846 **	0.708 **	0.846 **	0.717 **	−0.312	−0.333	−0.349
Feruloyl hexose		0.536 **	0.582 **	0.623 **	0.528 **	−0.402 *	−0.361	−0.400 *
Luteolin-7-O-glucuronide		0.593 **	0.685 **	0.354	0.822 **	0.438 *	0.454 *	0.498 *
Luteolin-7-O-glucoside		−0.142	0.432	−0.483 *	0.211	0.321	0.373	0.593 **
Rosmarinic acid		0.105	0.483 *	0.187	0.196	0.164	0.152	0.127
Salvianolic acid A isomer I		0.480 *	0.732 **	0.498 **	0.314	0.231	0.085	0.139
Salvianolic acid B		0.535 **	0.684 **	0.426 *	0.713 *	0.075	0.064	0.149
Total phenolic contents	F-C	0.741 **	0.765 **	0.580 **	0.822 **	−0.176	−0.174	−0.015
	HPLC	0.429 *	0.731 **	0.463 *	0.484 *	0.072	0.050	0.048

DPPH: 2.2-diphenyl-1-picrylhydrazyl; FRAP: ferric reducing antioxidant power; ABTS: 2.2′-azino-bis(3-ethylbenzothiazoline-6-sulfonic acid); ORAC: oxygen radical absorbance capacity; AChE: acetylcholinesterase; BChE: butyrylcholinesterase; Tyr: tyrosinase; F-C: Folin-Ciocalteu; HPLC: high performance liquid chromatography. ** Correlation is significant (*p* < 0.01). * Correlation is significant (*p* < 0.05).

## Data Availability

Not applicable.

## Supplementary Materials

The following are available online at https://www.mdpi.com/article/10.3390/antiox10040582/s1, Table S1: HPLC-HRMS data of identified phenolic compounds from *Lavandula pedunculata* subsp. *lusitanica* extracts, Table S2: summary of HPLC-HRMS parameters for quantification of phenolic compounds in *Lavandula pedunculata* subsp. *lusitanica* extracts, Figure S1: HPLC-HRMS extracted-ion chromatogram of phenolic compounds in *Lavandula pedunculata* subsp. *lusitanica* extracts obtained by ultrasound-assisted extraction for 60 min in combination with different NADES and conventional solvents. The compound name corresponding to each number of peaks can be consulted in Supplementary Table S1.
