# Supplementary material for: Ultrasonic-Assisted Extraction and Natural Deep Eutectic Solvents Combination: A Green Strategy to Improve the Recovery of Phenolic Compounds from Lavandula pedunculata subsp. lusitanica (Chaytor) Franco
Source: Antioxidants (Basel). 2021 Apr 9;10(4):582. doi: 10.3390/antiox10040582 (PMC8069433; doi:10.3390/antiox10040582)
Supplement: Supplementary file 1 [file antioxidants-10-00582-s001.pdf]

Ultrasonic-Assisted Extraction (UAE) and NADES Combination: A green strategy to Improve the Recovery of Phenolic Compounds from *Lavandula pedunculata* subsp. *lusitanica* (Chaytor) Franco

Inês Mansinhos[a,†], Sandra Gonçalves[a,†], Raquel Rodríguez-Solana[a,b], José Luis Ordóñez-Díaz[b], José Manuel Moreno-Rojas[b], Anabela Romano[a*]

[a]*MED – Mediterranean Institute for Agriculture, Environment and Development, Universidade do Algarve, Faculdade de Ciências e Tecnologia, Campus de Gambelas, 8005-139 Faro, Portugal*

[b] *Department of Food Science and Health, Andalusian Institute of Agricultural and Fisheries Research and Training (IFAPA), Avenida Menendez-Pidal, SN, 14004 Córdoba, Spain*

[†] *Equal authors*

* *Correspondence: aromano@ualg.pt; Tel.: +351289800910*

Supplementary information

Table S1. HPLC-HRMS data of identified phenolic compounds from *Lavandula pedunculata* subsp. *lusitanica* extracts.

Peak	Compound identity	Chemical formula	Theoretical exact mass [M-H]⁻ (*m/z*)	Delta ppm (error)	RT (min)	MSI MI level*
1	O-Caffeoylquinic acid[a]	$C_{16}H_{18}O_9$	353.0867	2.103	1.48	2
2	Gallic acid[b]	$C_7H_6O_5$	169.0131	-0.638	1.92	1
3	Vanillic acid[b]	$C_8H_8O_4$	167.0350	-2.606	2.37	2
4	Caffeic acid hexoside[a]	$C_{15}H_{18}O_9$	341.0867	-0.007	2.9	2
5	Caffeic acid hexoside[a]	$C_{15}H_{18}O_9$	341.0867	0.143	3.5	2
6	Chlorogenic acid (5-O-caffeoylquinic acid)[a]	$C_{16}H_{18}O_9$	353.0867	0.543	3.82	1
7	Fertaric acid[a]	$C_{14}H_{14}O_9$	325.0557	0.774	4.23	2
8	Caffeic acid[a]	$C_9H_8O_4$	179.0338	-0.604	4.59	1
9	Feruloyl hexose[a]	$C_{16}H_{20}O_9$	355.1028	1.187	6.60	2
10	Ferulic acid[a]	$C_{10}H_{10}O_4$	193.0495	-0.374	7.59	1
11	Luteolin-7-O-glucuronide[d]	$C_{21}H_{18}O_{12}$	461.0714	0.569	8.11	2
12	Luteolin-7-O-glucoside[d]	$C_{21}H_{20}O_{11}$	447.0929	1.660	8.12	2
13	Rosmarinic acid[a]	$C_{18}H_{16}O_8$	359.0761	0.488	9.57	1
14	Apigenin-7-O-glucoside[d]	$C_{21}H_{20}O_{10}$	431.0977	0.9594	9.72	2
15	Salvianolic acid A isomer I[a]	$C_{26}H_{22}O_{10}$	493.1129	0.508	10.11	2
16	Salvianolic acid A isomer II[a]	$C_{26}H_{22}O_{10}$	493.1129	0.358	10.97	2
17	Salvianolic acid I[a]	$C_{27}H_{22}O_{12}$	537.1027	1.499	11.13	2
18	Salvianolic acid B[a]	$C_{36}H_{30}O_{16}$	717.1450	-0.049	11.23	2
19	Apigenin-7-O-acetylglucoside[d]	$C_{23}H_{22}O_{11}$	473.1082	0.808	11.55	2
20	Herniarin[c]	$C_{10}H_8O_3$	175.0389	-0.429	12.14	2
21	Salvianolic acid A isomer III[a]	$C_{26}H_{22}O_{10}$	493.1129	-0.132	12.8	2
22	Luteolin[d]	$C_{15}H_{10}O_6$	285.0393	0.277	13.5	1
23	Naringenin[e]	$C_{15}H_{12}O_5$	271.0600	0.172	14.01	1
24	Apigenin[d]	$C_{15}H_{10}O_5$	269.0444	0.172	14.8	1

*Metabolite standards initiative metabolite identification (MSI MI) levels. Reference standards were available for all compounds identified at MSI MI level 1. Class of compounds: phenolic acids ([a]hydroxycinnamic acids and [b]hydroxybenzoic acids); [c]Coumarin derivative; flavonoids ([d]flavones and [e]flavanones).

Table S2. Summary of HPLC-HRMS parameters for quantification of phenolic compounds in *Lavandula pedunculata* subsp. *lusitanica* extracts.

Compound	Linear range (mg/L)	Intercept	Slope	R^2	LOD (mg/mL)	LOQ (mg/mL)
Rosmarinic acid	0.1-100	-103410	1598747	0.9992	0.06	0.19
Caffeic acid	0.1-10	1436398	3950274	0.9990	0.50	1.68
Ferulic acid	0.2-60	2487	9717	0.9995	0.37	1.25
Gallic acid	0.2-15	-330994	2547719	0.9998	0.00	0.01
Chlorogenic acid	0.2-15	-243627	1363025	0.9993	0.00	0.01
Luteolin	0.2-15	1464030	5454860	0.9990	0.29	0.96
Apigenin	0.2-15	185082	6649959	0.9995	0.00	0.01
Naringenin	0.2-15	-314843	2143048	0.9996	0.37	1.23
p-Coumaric acid	0.2-15	-2024	73034	0.9999	0.22	0.72

Figure S1. HPLC-HRMS extracted-ion chromatogram of phenolic compounds in *Lavandula pedunculata* subsp. *lusitanica* extracts obtained by ultrasound-assisted extraction for 60 min in combination with different NADES and conventional solvents. The compound name corresponding to each number of peaks can be consulted in Supplementary Table 1.

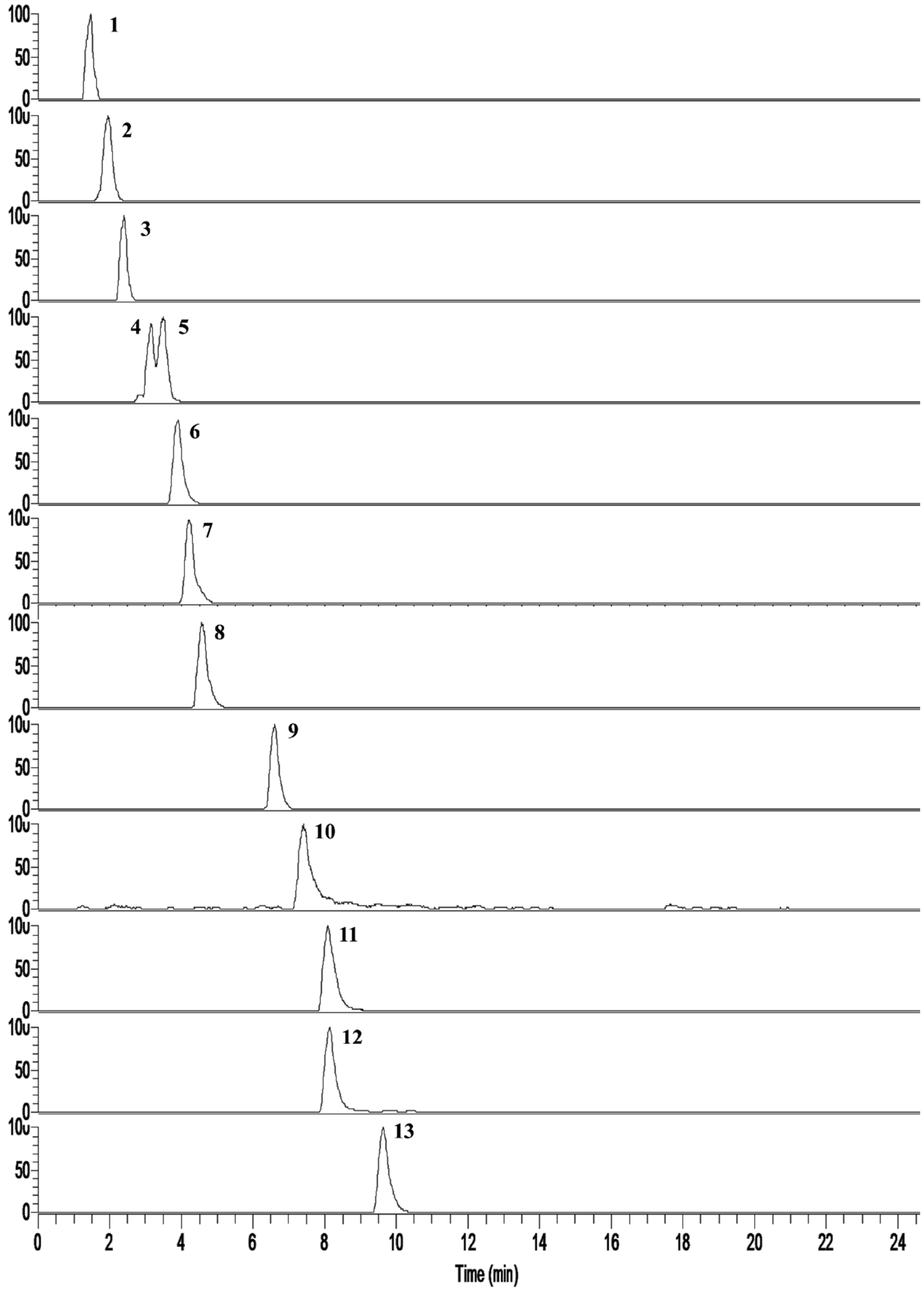

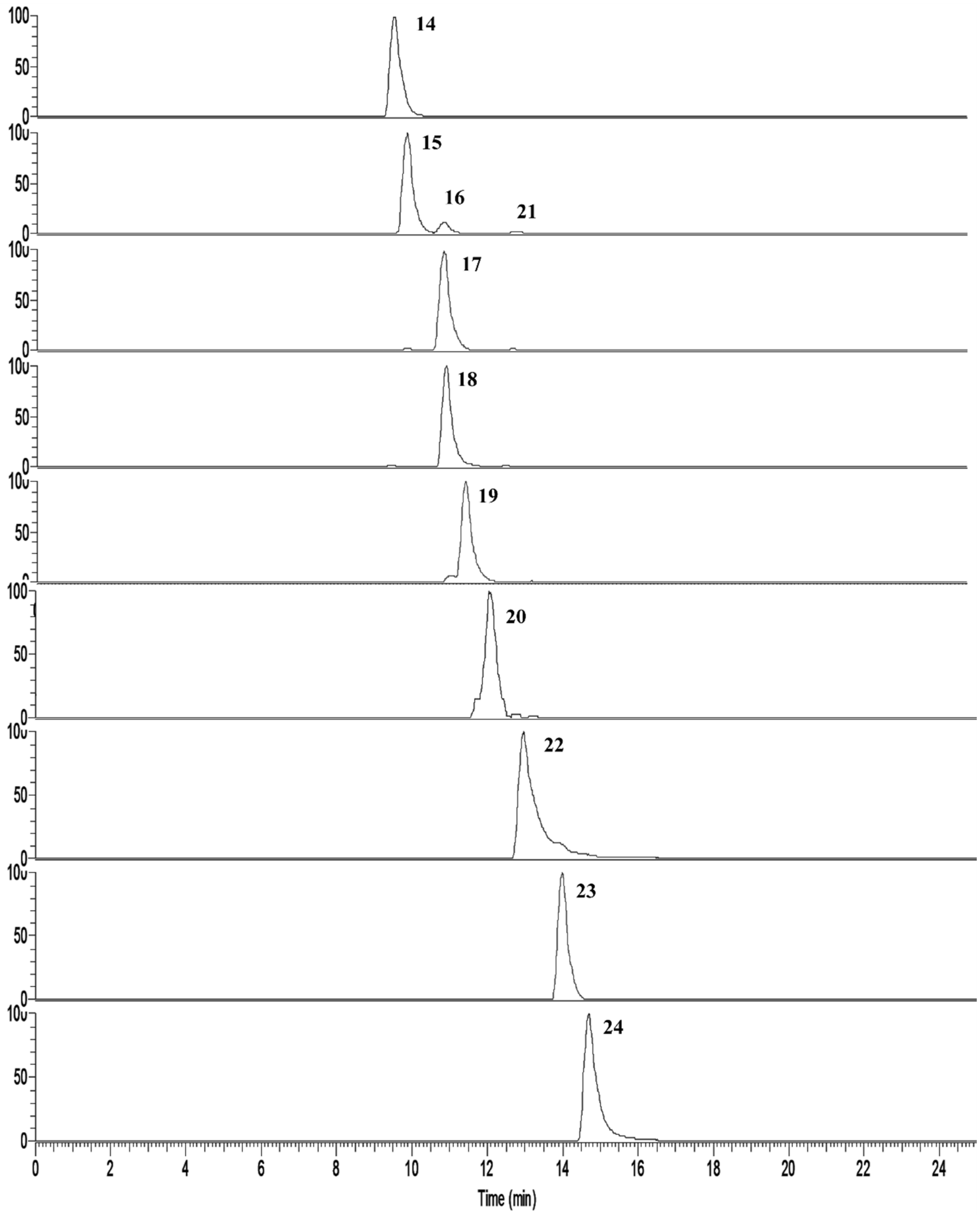
14
15
16
21
17
18
19
20
22
23
24
100
50
0
0
2
4
6
8
10
12
14
16
18
20
22
24
Time (min)
